# History of the London Royal Veterinary College, at St. Pancras

**Published:** 1818

**Authors:** James Carver


### HISTORY

OF THE

### *London Royal Veterinary College,*

AT ST. PANCRAS.

THE Royal Veterinary College, is an institution first established in the year 1792, at St. Pancras, near London. The public are indebted for this truly national foundation, to the discernment and patriotic exertions of the Agricultural Society of Oldham, in Hampshire. The first professor was Mr. St. Bell, a Frenchman, who had previously signalized himself in this country as a veterinary anatomist, by dissecting the famous horse Eclipse. The college is supported by an annual subscription. The annual contribution is two guineas, but the payment of twenty guineas at once, constitutes a subscriber for life. In some recent instances, the institution has shared the bounty of parliament; an immense saving resulted to the nation from the appointment of Veterinary Surgeons to the different regiments of British cavalry.

The views and objects of the college appear in the following statement, printed by the authority of the governors. The grand object, they observe, is the improvement of veterinary knowledge, in order to remedy the ignorance and incompetency of farriers, so long and so universally complained of. For this end, a range of stables, a forge, a theatre for dissections and lectures, with other buildings, have been erected; a gentleman of superior abilities has been appointed professor, with other requisite officers. The anatomical structure of quadrupeds, as horses, cattle, sheep, dogs, &c. the diseases to which they are subject, and the remedies proper to be applied, are to be investigated and regularly taught, by which means enlightened practitioners of liberal education, whose whole study has been devoted to the veterinary art in all its branches, may be gradually dispersed over the kingdom, on whose skill and experience confidence may securely be placed.

Pupils to the college, in addition to the lectures and instructions of the professors, and the practice of the stables, at present enjoy (from the liberality of some of the most eminent of the faculty) the advantages of free admission to their medical and anatomical lectures. These pupils, previous to leaving the college, are strictly examined by a medical committee, from whom they receive a proper certificate; and upwards of five hundred have been examined and approved, left the college, and are at this time practising in the different regiments of cavalry, and various parts of the country, with great success.

Subscribers have the privilege of sending their diseased animals to the college, without farther expense than that of daily food, and these in general form a sufficient number of patients for the practice of the professor and pupils. On fixed days, the professor prescribes for the animals belonging to the subscribers, who find it inconvenient to send them from home, provided the necessary medicines be furnished and compounded at the college. Subscribers' horses are there shod at the ordinary prices. His royal highness the commander in chief having been pleased to appoint a board of general officers to take into consideration the objects of this institution, and they have reported the continental loss to be very heavy, from the total ignorance of those who hitherto had the veterinary department in the army. This report his majesty approved, and henceforward to qualify for the military service, a veterinary surgeon must be provided with a regular diploma from the college. A number of gentlemen subscribers to the institution, attend once a fortnight to inspect the discipline of the stables, and see that the regulations are duly complied with.

THE PATRONS OF

### THE ROYAL VETERINARY COLLEGE

ARE AS FOLLOW, VIZ.

His Royal Highness PRINCE REGENT, President.

His Grace the DUKE OF NORTHUMBERLAND, K.G., F.R.S., F.A.S.

*Vice Presidents.*

LORD PERCY,
GEORGE, Earl of Morton, F.R.S., F.A.S.
GEORGE, Earl of Pembroke,
PHILIP, Earl of Chesterfield, F.R.S.
GEORGE, Earl of Macclesfield,
His Royal Highness DUKE OF KENT,
GEORGE, Earl of Warwick,
Sir T. C. BUNBURY, Bart., M.P.

GEORGE HOME SUMNER, Esq., M.P.
THOMAS PELL, Esq., F.A.S.
GRANVILLE PENN, Esq. F.A.S.

*Note.*—This last mentioned gentleman is of the Penn family of Pennsylvania, and is now living in England. He was the friend and patron of Mr. Charles Vial De St. Bell, the first professor of veterinary college; a great promoter of veterinary science, and the gentleman who laid the foundation stone of that institution.

The following gentlemen originally constituted the committee of examiners, for the purpose of granting diplomas to the pupils of the college, when sufficiently qualified to engage in practice.

DR. JOHN HUNTER,

G. FORDYCE,

BAILLIE,

BABBINGTON,

MR. ABERNETHY,

MR. HOULSTON,

CLINE,

A. COOPER,

HOME.

*The present Examining Committee are,*

H. CLINE, F.R.S., President,
Dr. BABBINGTON, F.R.S.
Dr. BAILLIE, F.R.S.
Sir EVERARD HOME, F.R.S.
J. ABERNETHY, Esq., F.R.S.
A. P. COOPER, Esq., F.R.S.
Dr. COOK,
Dr. PEARSON,
Dr. WILSON, F.R.S.
A. CLINE, Esq.
EDWARD COLEMAN, Professor,
WM. SEWELL, Esq. Assistant Professor, Treasurer and Secretary.

Among the improvements of these latter times, the extension of a regularly cultivated system of veterinary practice, and the attempts to rescue the superior class of domestic animals from the torturing hand of presumptuous ignorance, are not the least considerable, either in the view of humanity or life.

It is true, that during the various ages which have passed since the days of Columella, the number of writers treating on veterinary science, according to the best medical light which the time afforded has been considerable; but these works had never any very extensive circulation. Competent practitioners were wanting to put their precepts in force; and diseased animals were either totally neglected, or confided to the unmeaning and capricious efforts of the illiterate vulgar. Entirely to wipe away this opprobrium on humanity and common sense, must infinitely redound to the credit of the present times; and it is consoling to be able to announce, that attempts are daily making towards that beneficent end, by considerate and philanthropic characters in various parts of our own and neighbouring country.

Ancient prescriptions, and a false pride, among the medical faculty, compose the twofold cause which has hitherto deprived our domestic animals of the benefits and comforts of regular assistance. Cattle have always been doctored in every country, either by their attendants, or by men pretty nearly on a level with those, in point of education, who, on the strength of having learned to perform the most simple and common operation, and from the want of able proficients, have undertaken the arduous task of prescribing medicine. We need not wonder, that in former times such professors were held duly qualified, since men impartially committed their own persons to the hands of ignorant barber surgeons; and since so many absurdities of equal magnitude subsisted, which, like spectres and ghosts, have vanished at the approach of modern light. But it may well be thought surprizing, that in this discerning age, when a liberal education is universally acknowledged to be absolutely necessary to the acquisition of medical science, that an illiterate farrier should be trusted in the cure of diseases. Precisely the same studies, physiological, anatomical. and medical, are requisite for the veterinarian as the human practitioner. The animal economy, in its manifold relations, is generally fundamentally the same in men and beasts, and governed by the same laws; the same materia medica is, in a great degree, applicable to both. But the greatest skill is requisite to form a judgment of the symptoms of diseases in brutes, from their inability to describe their own feelings, and the consequent uncertainty of their pathology.

Can there be a greater burlesque, than the supposition of a man's ability to prescribe physic for a horse, merely because he knows how to groom and shoe him? or might we not also, with equal reason, employ our own shoemaker to take measure of our health? The plea of experience is futile, from the utter inability (prima facie) of illiterate and uninformed men to investigate the principles of science, and their total want of opportunity to acquire, by rote, a rational system of practice. The whole stock of medical knowledge of these practitioners, usually consists in a certain number of receipts, derived from their masters or fathers, and which they continually ring the changes, in all cases, right or wrong; and so fiercely are they bigoted to their own peculiar nostrums, that they are totally incapable of all advice or improvement—the common and unavoidable fate of confirmed ignorance; since it is the highest point of knowledge to know that we still need information. They sometimes cure by luck, seldom from knowledge, but often kill by regularly adapted process. How often has the miserable patient's shoulder been pegg'd, and blown, and bored, by way of punishment, for the folly of getting himself strained in the back sinews of the leg, or coffin joint! How many pleuretic horses have been killed outright by ardent spicy drenches, which probably might have cured the cholic! How many have been rendered incurably lame, from the patent shoe being affixed to the wrong foot!—Let not the reader suppose these to be mere flourishes, applied to the generality of farriers within my knowledge: I aver them, on the experience of many years, to be literal truths; and by the tenor of them, he may judge of the majority of that faculty throughout Europe. Into such hands do we commit our distempered animals, which have it not in their power to reproach us with their accumulated sufferings; mankind from prejudice, indolence, or want of feeling, neglecting those creatures which they can purchase with their money.

It has been supposed that veterinary writers have been wanting. It was many years ago discovered in France, that the best remedy for this defect, and the only adequate method for the general diffusion of veterinary knowledge, and the rearing of a sufficient number of persons properly qualified in that line, would be to erect public seminaries expressly dedicated to the purpose.

We, of this country, came (late indeed), into the same salutary measures; and a Veterinary College, as an hospital for cattle, has been established in London, and others in various parts of the kingdom. The propriety of these steps, and the benefits derived from them, must be obvious in the extension of veterinary knowledge and the increase of practitioners.

Public institutions, provided they are not unduly favoured with exclusive privileges, or armed with coercive and restrictive powers, are ever most efficacious and contributory to the advancement of science. The scattered rays of knowledge are, by joint and public means, best collected into a common focus or centre; whence they are, with more ease and expedition, diffused and circulated throughout the whole body of the commonwealth. The Veterinary College has also adopted a very judicious method of disseminating the true principles of shoeing, by erecting forges in different quarters of the metropolis, where all persons may at any time have their horses shod at the common price charged to subscribers. Prejudice, I know, on more important occasions, has often been trumpeted forth as not only harmless, but beneficial among men; which indeed would be just, were there any general utility in the continuance of ancient abuses. It is the grand business of philosophy to provide a counterblast for these interested or ingnorant trumpeters.

It has already been asked, for the advocates of our shoeing and sow-gelding doctors, how they came to suppose that less medical knowledge would suffice to prescribe for the brute than for the human animal, who can orally depict his own feelings, and verbally assist the physician in forming a correct judgment of his disease. They seem to act upon the strange supposition, that it is much easier for an illiterate man to penetrate at once, as it were, by INTUITION, into the arcana of the sciences, than for a learned or well informed man to render himself skilful in the nature and management of horses. Can a man be the worse farrier for having learned the necessity of making constant observation of his own, instead of acting by rote, and being guided by a few arbitrary receipts, for knowing the nature of the medicines he prescribes, the anatomy and animal functions of the horse, and for making all such knowledge his study?

In fine, all at this moment appears obscured or bewildered, by the ill-placed confidence of the owners of cattle upon the blacksmith of the parish; upon illiterate and conceited grooms, stupid and listless shepherds; or upon a set of men infinitely more dangerous than all the rest, who, arrogating to themselves the style of doctors, ride about from town to town, and from village to village, distributing their nostrums, compounded of the refuse and vapid scraps of druggists' shops, to the destruction of thousands, whose varied disorders they treat alike, neither consulting nature or art for the cause or the effect. Miserable animal! bereft of speech, thou canst not complain; when, to the disease with which thou art afflicted, excruciating torments, are superadded by the ignorant efforts of such men, who, at first sight, and without any investigation to lead them to the source of thy disorder, pronounce a hackneyed, common-placed opinion on thy case, and then proceed, with all expedition, to open thy veins, lacerate thy flesh, cauterize thy sinews, and drench thy stomach with drugs, adverse in general to the cure they engage to perform.

Opposed to this barbarous and noxious practice, let us turn our eye to that of the Veterinary Physician and Surgeon.

We shall not find him occupying the attention of his auditors with the accounts of miraculous cures he never performed,—or, under the mask of sullen ignorance, endeavouring to attract confidence; we shall not see him armed at all points with phlemes, rowelling knives, and cauterizing irons, to rack and torment his suffering patients; or with drenches or balls, to obstruct the efforts of nature. We shall see him with a cautious eye and tender hand, surveying and examining, with discretion and judgment, into the case before him; and, as far he can attain information from those who bring the animal to him, we shall find him an anxious and patient inquirer; proceeding to explore all the external signs, and to observe with great minuteness every symptom which presents itself; and if he finds them so complicated that he cannot with certainty proceed to give an opinion, he will wait until some new, or more distinct appearances come to his assistance. If, however, these signs should not show themselves, to give effect, he will then apply to the only resource left him—that of compelling nature to develop herself; or, at least, to show some indications. This he accomplishes through the means of medical aid, administered in proper quantities, which, by increasing more or less sensibly the disease, produces some discovery of its tendency.

Now that witches and ghosts of all kinds are flitting apace off the scenes, it is full time for men to lay aside the expectation of all other uncaused effects. On these topics a celebrated veterinary writer dwells, with peculiar force of illustration, as he says, " from a motive of justice, on account of the irrational prejudice of too many persons concerning the Veterinary College."

" Enjoying a public institution in the metropolis," says he, " where veterinary science, in all its branches, is regularly taught and practised, it remains for those who interest themselves in the safety and well being of our domestic animals, to devise and recommend the most proper and expeditious methods of a general diffusion of these benefits throughout the country. The farriers of London were advised, by persons of influence, to allow their sons and apprentices to attend the college lectures which are given, and which indeed is practised by several of good repute. Those gentlemen of the medical profession, attending the London hospitals, whose destination is for country practice, will surely perceive great probable advantage in the acquisition of veterinary knowledge, even if they have no present intention to profess that branch of medicine. Business, as is sometimes the case with young practitioners, may run short at the outset, and the leisure time might be both honourably and profitably employed in veterinary practice. Such meritorious and humane occupation, could not possibly injure the medical character of a medical gentleman in these enlightened times; on the contrary, it would be more probable to procure him connexions of the most valuable sort—and might be his passport and introduction to the families of medical men."

Thus far we have stated the opinions of a writer truly ingenious, and most deservedly popular. Just, however, as are the encomiums of this useful institution at an early period of its existence, yet we are bound more especially to acknowledge the extraordinary progress which this institution afterwards made (and is now making) under its present enlightened and truly ingenious professor Mr. Coleman. This gentleman, to a natural taste for these investigations, united a profound knowledge of his profession, as an anatomist and surgeon—a foundation on which the Veterinary Science could not but be erected with singular advantage. That this has actually been the case, our readers must be aware that from the report of 1814, published in London, brought over by Mr. Carver, that not less than six hundred students have passed at this institution, who are now attached to the different regiments of British cavalry, and also practising in various parts of the united kingdom; besides the different articles in which Mr. Coleman's name and writings have necessarily been brought forward; for which reason we close the present article without entering on those particulars which it would, otherwise, have been our indispensable duty to have stated.

